# Real-Time Decreased Sensitivity to an Audio-Visual Illusion during Goal-Directed Reaching

**DOI:** 10.1371/journal.pone.0008952

**Published:** 2010-01-29

**Authors:** Luc Tremblay, Thanh Nguyen

**Affiliations:** Faculty of Physical Education and Health, University of Toronto, Toronto, Ontario, Canada; University of Queensland, Australia

## Abstract

In humans, sensory afferences are combined and integrated by the central nervous system (Ernst MO, Bülthoff HH (2004) Trends Cogn. Sci. 8: 162–169) and appear to provide a holistic representation of the environment. Empirical studies have repeatedly shown that vision dominates the other senses, especially for tasks with spatial demands. In contrast, it has also been observed that sound can strongly alter the perception of visual events. For example, when presented with 2 flashes and 1 beep in a very brief period of time, humans often report seeing 1 flash (i.e. fusion illusion, Andersen TS, Tiippana K, Sams M (2004) Brain Res. Cogn. Brain Res. 21: 301–308). However, it is not known how an unfolding movement modulates the contribution of vision to perception. Here, we used the audio-visual illusion to demonstrate that goal-directed movements can alter visual information processing in real-time. Specifically, the fusion illusion was linearly reduced as a function of limb velocity. These results suggest that cue combination and integration can be modulated in real-time by goal-directed behaviors; perhaps through sensory gating (Chapman CE, Beauchamp E (2006) J. Neurophysiol. 96: 1664–1675) and/or altered sensory noise (Ernst MO, Bülthoff HH (2004) Trends Cogn. Sci. 8: 162–169) during limb movements.

## Introduction

The natural world stimulates our many senses, which provide a unique percept through multisensory combination and integration [1]. Using various methods, multisensory research has repeatedly shown that certain modalities can alter the perception of other modalities [2]–[6]. For example, it has been reported that the perceived number of brief visual flashes is influenced by the number of short accompanying beeps [7] (e.g. 2 flashes accompanied with 1 beep often yields the perception of 1 flash: i.e. fusion illusion). Further, the presence of the illusory experience is associated with changes in primary visual cortex activity [8]. This audio-visual illusion also demonstrates the dominance of audition in a temporally demanding task. In contrast, we know from other multisensory integration studies that vision predominantly influences audition in spatially demanding tasks [2]–[4], [9]. However, the influence of limb movement on multisensory integration is not known. Indeed, in multisensory studies, either the stimuli were presented to a participant at rest or the influence of any required motor responses on the investigated perceptual processes was not assessed.

Neural-behavioral research has accumulated evidence that vision is an important source of afferent information for the planning and control of goal-directed movements [10]. More importantly, it has also been shown that action can influence the perception of non-visual events [11], [12]. Specifically, the production of a voluntary movement can modulate the detection of a tactile stimulation (i.e. action onset decreases the perception of a brief finger stimulation [11], [12]). It has been suggested that such “gating” of tactile information is associated with modulation of central nervous system activity at the pre-cortical level [13]. Thus, if producing a voluntary movement reduces the tactile detection threshold, it is possible that the processing of other sensory inputs—relevant to the experimental task–increases.

This study aimed to demonstrate that a spatially demanding goal-directed action modulates the relative processing of audition and vision in real-time. Participants (n = 14) quickly moved their right index finger towards a small visual target and the presentation of 1 or 2 flashes accompanied with 1 or 2 auditory beeps (i.e. audio-visual illusion stimuli [5]) started at 0 ms, 50 ms, 100 ms, 150 ms, or 200 ms relative to movement onset. We hypothesized that the perceptual effects of the audio-visual illusion would be influenced by the real-time characteristics of the voluntary action. Such result would support the idea that cue combination [1] can be modulated in real-time during voluntary movements.

## Results and Discussion

**Figure 1:**
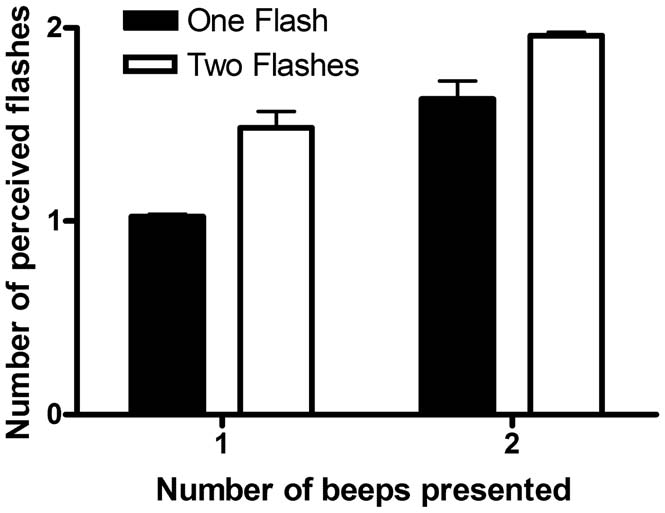
Mean number of perceived flashes as a function of the number of flashes and beeps. There was a main effect for flash (*F* (1, 13) = 31.63, *p*<0.001) and beep (*F* (1, 13) = 64.10, *p*<0.001).

When 1 flash and 2 beeps were presented, participants perceived 2 flashes (i.e. fission illusion [7]) on 63% of the trials (see Table 1). When 2 flashes and 1 beep were presented, participants perceived 1 flash (i.e. fusion illusion [7]) on 52% of the trials (see Table 1). Thus, our methodology reproduced both the fission [5], [6] and fusion [7] illusions (see Figure 1).

**Table 1:** Proportion of trials where an illusion was perceived (and standard error of the mean) as a function of the experimental conditions.

	0 ms	50 ms	100 ms	150 ms	200 ms
**1Flash-1Beep**	2% (1%)	1% (1%)	3% (1%)	4% (2%)	2% (1%)
**1Flash-2Beep**	62% (9%)	65% (9%)	65% (9%)	64% (9%)	59% (10%)
**2Flash-1Beep**	57% (9%)	44% (8%)	44% (8%)	52% (10%)	63% (8%)
**2Flash-2Beep**	2% (1%)	1% (1%)	4% (2%)	6% (3%)	8% (3%)

**Figure 2:**
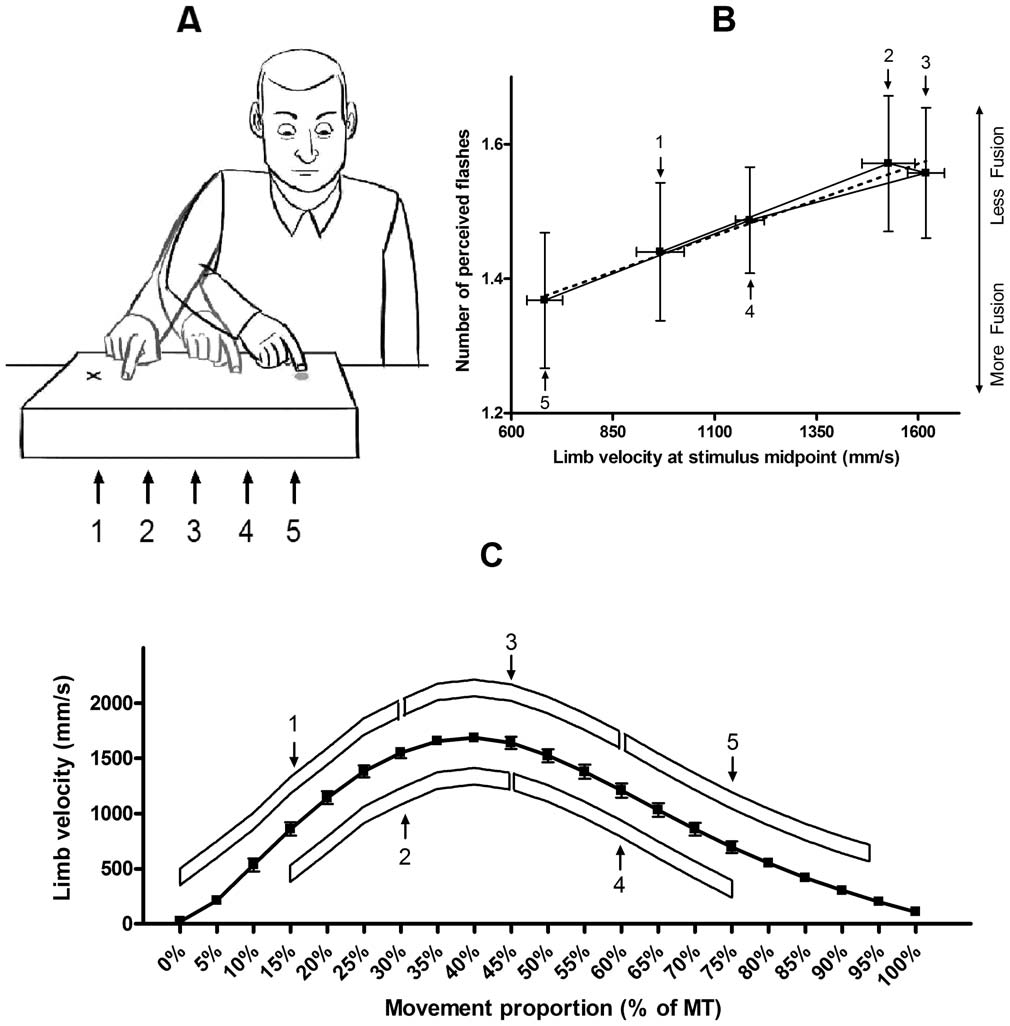
Experimental Task, Limb Velocity vs. the Fusion Illusion, and Average Velocity Profile. Panel A: Depiction of the experimental task. Panel B: Mean number of perceived flashes for the 2 flashes and 1 beep condition at the different stimulus midpoints. Panel C: Average limb velocity profile with depiction of stimuli presentation (white boxes) and stimuli midpoints (arrows). The stimulus onset conditions are numbered as follows: 1 = 0 ms, 2 = 50 ms, 3 = 100 ms, 4 = 150 ms, and 5 = 200 ms relative to movement onset. Error bars represent standard error of mean and dashed line represents best line of fit.

In addition to replicating the audio-visual illusion, we found that participants were less influenced by the illusion when their limb was moving at high velocities. When 2 flashes accompanied 1 beep, participants reported 1 flash (i.e. fusion illusion) more often in the early and late portions of the movement (i.e. 0 ms and 200 ms conditions corresponding to the lowest limb velocities) than in the 50 ms and 100 ms conditions (i.e. at the highest limb velocities) (*p*s<.02). As such, the fusion illusion was experienced 57% and 63% of the time in the 0 ms and 200 ms conditions respectively while it was only reported 44% of the time in the 50 ms and 100 ms conditions (see Table 1). When contrasting limb velocity at stimulus midpoint (i.e. 50 ms after stimulus onset) with the number of perceived flashes in the 2 flashes with 1 beep condition, we observed**—**across all experimental trials presenting 2 flashes and 1 beep**—**that the fusion illusion was linearly reduced as a function of limb velocity (see Figure 2).

Our results show that the fusion illusion occurred less often at the high than the low velocity stages of the limb trajectory. While neural-behavioural, psychophysical and neuroimaging studies support the idea that different modalities are combined and integrated [14], this study shows that the mere fact of moving a limb influences such multisensory integration processes in real-time. Possible explanations for these results include sensory “gating” mechanisms [11], [12] and/or varying sensory noise levels [1] associated with goal-directed behaviors. That is, the altered relative contribution of vision and audition during voluntary action could be associated with reduced processing of non-visual cues during a visually-guided task (i.e. “gating”) [11], [12] and/or increased visual processing caused by larger contrasts of the limb position on the retina between visual samples (i.e. higher visual signal-to-noise ratio at high limb velocities) [1].

In terms of the sensory “gating” perspective [11], [12], one possible explanation is that the central nervous system modulates its use of sensory information in real-time, as a function of the relevance of the afferent cue. Chapman and colleagues observed that tactile cues were less likely to be detected in close temporal proximity of the onset of a finger movement [11], [12]. This decreased tactile sensitivity could be explained by an increased sensitivity to visual cues, which were relevant to the task at hand. In the present study, we purposefully employed a spatially demanding goal-directed action that requires extensive use of visual information [10]. Using such task, it is reasonable to suggest that the central nervous system modulated its use of visual information in real-time as a function of the relevancy of the visual cue. Indeed, high limb velocities can elicit stronger visual signals for the control of goal-directed actions than low limb velocities.

At high limb velocities, two subsequent visual samples provide greater differences in the position of the limb on the retina (i.e., stronger signal) than at low limb velocities. If the noise present in the visual cues provided to the central nervous system is relatively stable, then the signal-to-noise ratio is modulated in real-time as a function of limb velocity during goal-directed action. Such signal-to-noise ratio is known to influence multisensory cue combination and integration [1], [15]. Thus, our study suggests that optimal cue combination and integration could be modulated in real-time during goal-directed movements—which is not mutually exclusive with sensory “gating” [11], [12].

In summary, our observations demonstrate the real-time modulation of visual perception during the production of voluntary movements. Thus, the relative contribution of visual and auditory information to our percept is not held constant throughout a goal-directed movement, but is at least modulated as a function of limb velocity.

## Materials and Methods

Fourteen right-handed persons (5 females) with normal to corrected-to-normal vision and hearing were recruited from the University of Toronto community (mean age: 23.8 years, SD = 4.4). This protocol was approved by the University of Toronto Research Ethics Board and is also in accordance to the standards outlined in the 1964 Declaration of Helsinki. Written informed consent was obtained prior to any experimental involvement.

The task was performed using an aiming console (see Figure S1) equipped with 2 LEDs (target: green LED; flash: red LED) and a piezoelectric buzzer (2900 Hz). The position of an infra-red emitting diode (IRED) sampled at 250 Hz (Opototrak Certus, Northern Digital Inc.) and a custom-made program (MatLab, The Mathworks Inc.) were used to track the participant's movements and control stimuli presentation, respectively.

After placing the IRED on the tip of the right index finger, participants sat down and were asked to reach from a home position to a target (30 cm movement amplitude), which was aligned with their mid-saggital axis (see Figure 2). In a familiarization phase, participants were taught how to complete the movement within approximately 290 to 350 ms. In the experimental phase, 1 or 2 red flashes accompanied with 1 or 2 auditory beeps were also presented below the green target LED at 0 ms, 50 ms, 100 ms, 150 ms, or 200 ms after movement onset (i.e. 2 flash×2 beep×5 time). Each condition was presented 12 times each (i.e. 240 trials) in a pseudo-random fashion without repeating a condition more than 3 times in a row. Stimulus duration was 24 ms and stimulus onset asynchrony was 36 ms (see Figure S2). Participants were asked to report the number of flashes perceived after each trial (i.e. 1 or 2 flashes).

ANOVAs were performed on the mean number of perceived flashes. Alpha level was set at .05 and Tukey HSD post hoc procedures were preformed on the significant main effects and interactions, when appropriate.

## Supporting Information

Figure S1 Aiming console. Board viewed from the participant's side of the table. The custom built console, measuring 50 cm wide×27.5 cm deep×8.5 cm high, was placed 36 cm from the edge of the table from where participants were seated. A green target LED was located 30 cm to the left of home position. The red stimulus LED was located 6 cm below the target. The piezoelectric auditory stimulus was located 7 cm below the target, within the console. Participants aligned their mid-saggital plane with the target.
(7.52 MB TIF)

Figure S2 Temporal profile of stimuli. Profile of two-cue stimulus presented to one modality and one-cue stimulus presented to another modality.
(0.39 MB TIF)

## References

[1] Ernst MO, Bülthoff HH (2004). Merging the senses into a robust percept.. Trends Cogn Sci.

[2] Howard IP, Templeton WB (1966). Human spatial orientation. (Wiley)..

[3] McGurk H, MacDonald J (1976). Hearing lips and seeing voices.. Nature.

[4] Rock I, Victor J (1964). Vision and touch: An experimentally created conflict between the two senses.. Science.

[5] Shams L, Kamitani Y, Shimojo S (2000). What you see is what you hear: sound induced visual Xashing.. Nature.

[6] Shams L, Kamitani Y, Shimojo S (2002). Visual illusion induced by sound.. Brain Res Cogn Brain Res.

[7] Andersen TS, Tiippana K, Sams M (2004). Factors influencing audiovisual fission and fusion illusions.. Brain Res Cogn Brain Res.

[8] Watkins S, Shams L, Tanaka S, Haynes JD, Rees G (2006). Sound alters activity in human V1 in association with illusory visual perception.. Neuroimage.

[9] Witten IB, Knudsen EI (2005). Why seeing is believing: merging auditory and visual worlds.. Neuron.

[10] Elliott D, Helsen WF, Chua R (2001). A century later: Woodworth's (1899) two-component model of goal-directed aiming.. Psychol Bull.

[11] Chapman CE, Beauchamp E (2006). Differential controls over tactile detection in humans by motor commands and peripheral reafference.. J Neurophysiol.

[12] Williams SR, Chapman CE (2002). Time course and magnitude of movement-related gating of tactile detection in humans. III. Effect of motor tasks.. J Neurophysiol.

[13] Jiang W, Chapman CE, Lamarre Y (1991). Modulation of the cutaneous responsiveness of neurones in the primary somatosensory cortex during conditioned arm movements in the monkey.. Exp Brain Res.

[14] Liu Q, Qiu J, Chen A, Yang J, Zhang Q (2007). The effect of visual reliability on auditory-visual integration: an event-related potential study.. Neuroreport.

[15] Ernst MO, Banks MS (2002). Humans integrate visual and haptic information in a statistically optimal fashion.. Nature.

