# Supplementary figures and images for: Real-Time Decreased Sensitivity to an Audio-Visual Illusion during Goal-Directed Reaching
Source: PLoS One. 2010 Jan 29;5(1):e8952. doi: 10.1371/journal.pone.0008952 (PMC2813281; doi:10.1371/journal.pone.0008952)

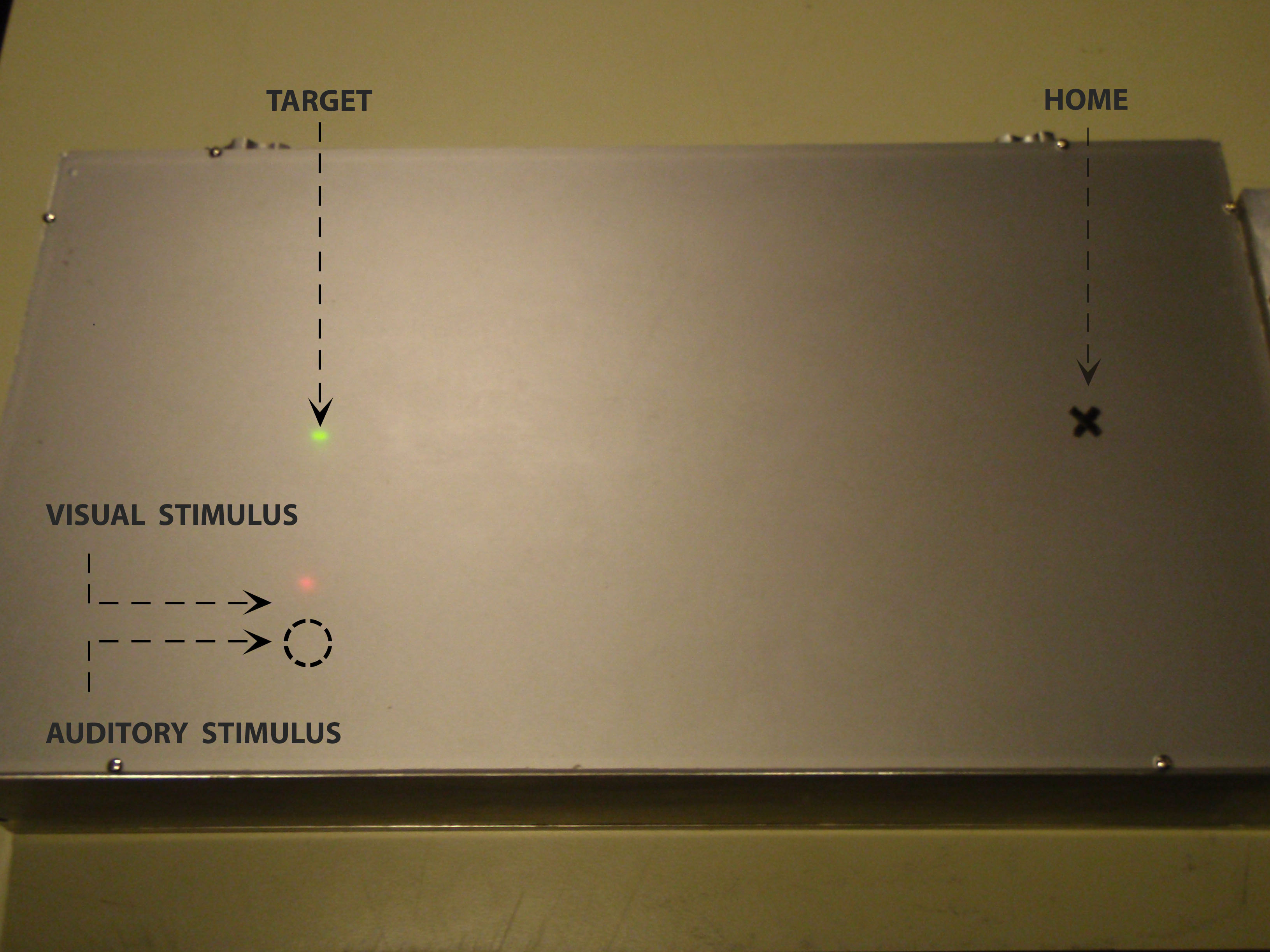

Supplement: Figure S1 — Aiming console. Board viewed from the participant's side of the table. The custom built console, measuring 50 cm wide×27.5 cm deep×8.5 cm high, was placed 36 cm from the edge of the table from where participants were seated. A green target LED was located 30 cm to the left of home position. The red stimulus LED was located 6 cm below the target. The piezoelectric auditory stimulus was located 7 cm below the target, within the console. Participants aligned their mid-saggital plane with the target. (7.52 MB TIF) [file pone.0008952.s001.tif]

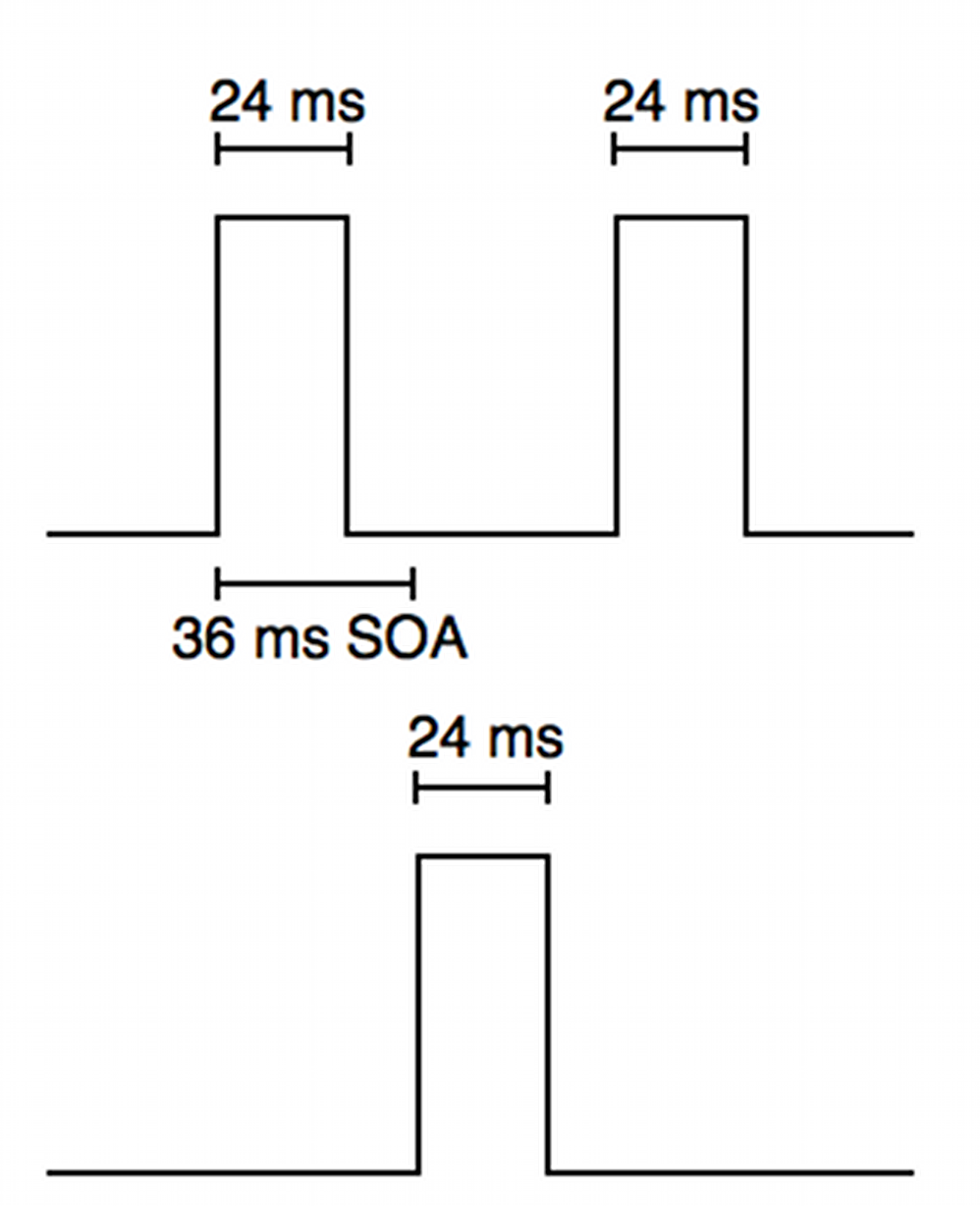

Supplement: Figure S2 — Temporal profile of stimuli. Profile of two-cue stimulus presented to one modality and one-cue stimulus presented to another modality. (0.39 MB TIF) [file pone.0008952.s002.tif]
